# Pretemporal Transcavernous Approach to the Intracavernous Internal Carotid Artery: A Microsurgical Anatomy Study

**DOI:** 10.7759/cureus.96987

**Published:** 2025-11-16

**Authors:** Antonio García-López, Sofía Sotos Picazo, Claudio Piqueras Pérez

**Affiliations:** 1 Neurosurgery, Virgen de la Arrixaca University Clinical Hospital, Murcia, ESP

**Keywords:** cavernous sinus, intracavernous internal carotid artery, microsurgical anatomy, pretemporal transcavernous approach, vascular neurosurgery

## Abstract

Background

The cavernous sinus (CS) is a complex and deep-seated structure that requires extensive anatomical knowledge and microsurgical training. Direct microsurgical repair of the intracavernous internal carotid artery (IC-ICA) pathologies has been largely avoided, favoring other treatment options, such as endovascular or bypass procedures. Expanding our understanding of microsurgical anatomy could improve the direct surgical options for IC-ICA pathologies.

Objective

We describe the IC-ICA access triangles and perform a quantitative comparative analysis.

Methods

Thirteen cadaveric heads were subjected to a pretemporal transcavernous approach. The clinoidal triangle (CT), infratrochlear triangle (ITT), and anteromedial triangle (AMT) were exposed. Triangle areas and IC-ICA lengths exposed within each triangle were determined under the microscope.

Results

A total of 25 CSs were successfully analyzed. The triangle areas measured 56.3 ± 29.4 mm^2^, 48.9 ± 27.3 mm^2^, and 44.1 ± 19.6 mm^2^ for ITT, AMT, and CT, respectively (p=0.403). The IC-ICA lengths exposed measured 11.3 ± 4.3 mm, 7.4 ± 3.9 mm, and 7.7 ± 3.5 mm for ITT, AMT, and CT, respectively (p=0.002).

Conclusions

The ITT is a CS window that provides wide access to the IC-ICA. This window can be used to directly repair various IC-ICA pathologies, such as aneurysms or carotid cavernous fistulas.

## Introduction

The cavernous sinus (CS) is a dural venous sinus located within the middle cranial fossa, specifically in the parasellar region. It contains the internal carotid artery (ICA) (C3 segment) and cranial nerve VI. The lateral wall contains cranial nerves III, IV, V1, and V2 [1]. In addition, the CS has a complex venous network. Because of this complex neurovascular structure, which is also deep-seated, the CS has been considered a “no man’s land.” Since Parkinson’s description of the microsurgical anatomy and approach to the intracavernous ICA (IC-ICA) [2], most neurosurgeons have avoided the CS approach because of the complications described [3-5].

Currently, owing to the risks of CS surgery, endovascular techniques have become the primary treatment modality for IC-ICA pathologies, such as aneurysms or carotid cavernous fistulas (CCFs) [6]. Endovascular options are less invasive than microsurgery but are usually less durable, require more frequent retreatments, fail to address the compressive effects of the pathology on cranial nerves, and frequently require chronic antiplatelet therapy [6-11].

In our opinion, the CS can be safely navigated with proper microsurgical training and extensive anatomical knowledge [12-20]. Microsurgical dissection of the CS is an excellent method to treat vascular and tumoral pathologies of the skull base [21]. The pretemporal transcavernous approach offers, with proper training, a safe, reproducible, fast and wide exposure of CS [14,15,21]. Furthermore, this approach is performed “under the brain” and provides a direct skull base view with minimal or no brain retraction.

This study describes, in detail, the microsurgical anatomy of the CS and the relation between the nerves and IC-ICA. The purpose of this study is to define the optimal window to access the IC-ICA to improve microsurgical solutions for pathologies of this carotid segment.

## Materials and methods

The study was conducted in the Anatomy Laboratory of the University of Murcia (Murcia, Spain) in February 2024. The laboratory adhered to local laws and obtained the necessary permissions to work with human cadavers. IRB approval was not required for this study, as it did not involve human participants or identifiable data. All cadavers used in this study were donated to the Department of Anatomy at University of Murcia in accordance with institutional and national ethical standards.

The CS was studied using a pretemporal transcavernous approach. Thirteen cadaveric heads, embalmed in a customized alcohol-based solution, were infused with colored silicone into the arteries and veins. Each head was secured in a three-pin Mayfield clamp (Integra LifeSciences, New Jersey, US), turned 30° to the contralateral side, and slightly extended. A curved incision was made just anterior to the tragus, extending from the superior temporal line to the mid-pupillary line behind the hairline. The cutaneous and muscle flaps were reflected in two layers using an interfascial technique (Yasargil flap), allowing for greater exposure of the temporal and pretemporal regions than the standard pterional approach. A frontotemporal craniotomy (Stryker Core, Wisconsin, USA) was performed, exposing the floor of the middle fossa and roof of the orbit. The dura was separated from the bone under a microscope (Carl Zeiss Prescott, Oberkochen, Germany) to visualize the foramen spinosum, foramen ovale, foramen rotundum, and superior orbital fissure. After cutting the middle meningeal artery, the posterior third of the roof and lateral orbital wall were drilled and removed to expose the meningo-orbital band. After cutting the meningo-orbital band, the dura propria of the temporal lobe was dissected from the lateral wall of the CS. Dissection continued until the V1, V2, and V3 nerves, as well as the petrous apex with the greater superficial petrosal nerve, were visualized. The optic roof and the optic strut were drilled to perform the extradural anterior clinoidectomy. Using microsurgical techniques, the CS nerves were skeletonized, and the IC-ICA was exposed.

The areas of the following spaces were measured under the microscope: the clinoidal triangle (CT) (Dolenc), infratrochlear triangle (ITT) (Parkinson), and anteromedial triangle (AMT) (Mullan) [22]. Furthermore, the IC-ICA length exposed in each triangle was also measured.

IBM SPSS Statistics for Windows, Version 28 (Released 2021; IBM Corp., Armonk, New York, United States) was used for statistical analysis. Continuous variables were described as mean ± standard deviation. The non-parametric Kruskal-Wallis test was used to compare means after verifying the non-normal distribution of the quantitative data using the Kolmogorov-Smirnov test. Statistical tests were performed with a type I error rate of 5%.

## Results

**Figure 1:**
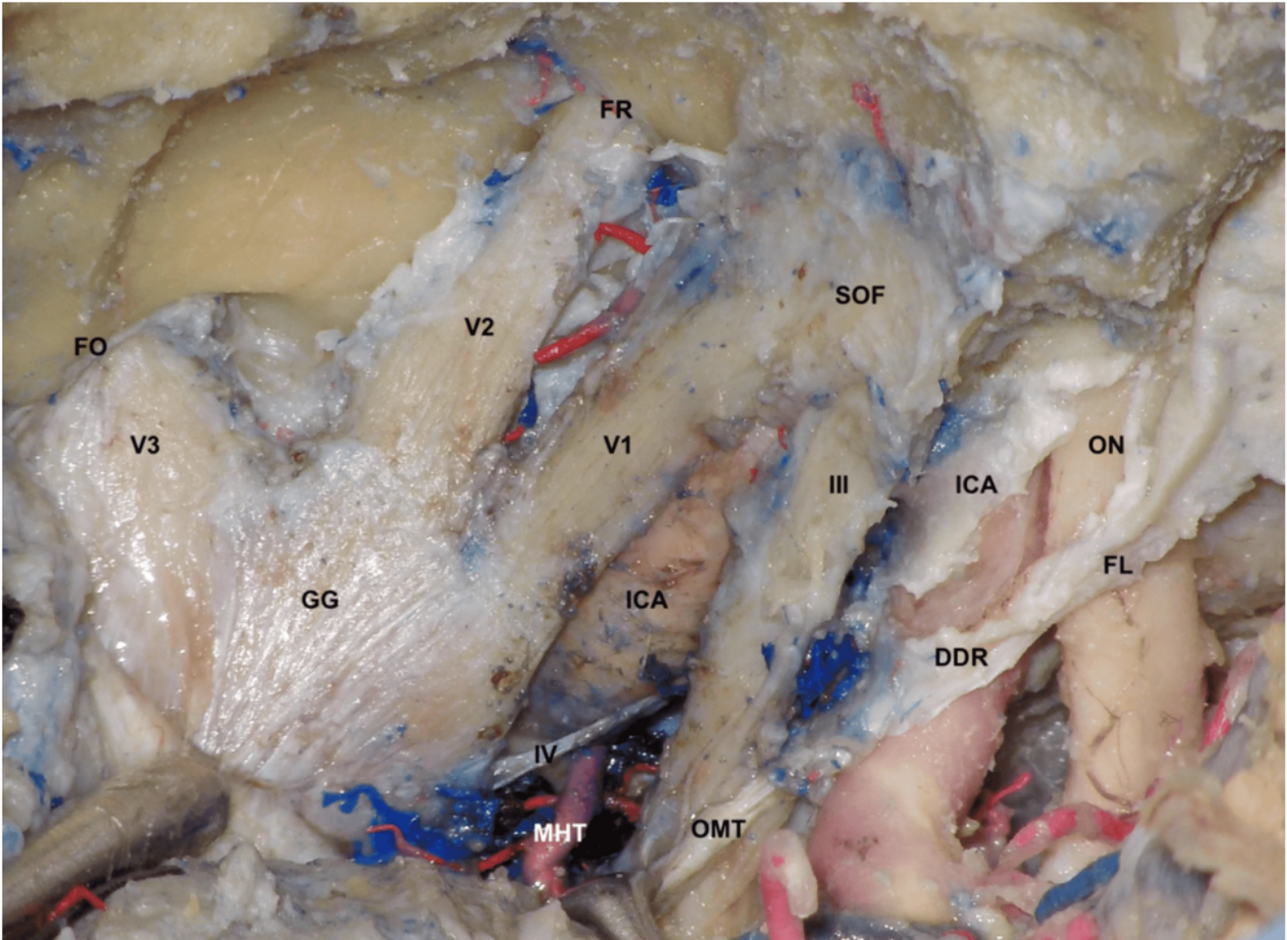
Microsurgical exposure of the left CS through a pretemporal transcavernous approach The nerves were skeletonized, and the cavernous ICA was exposed. ON: optic nerve; FL: falciform ligament; ICA: internal carotid artery; DDR: distal dural ring; III: oculomotor nerve; OMT: oculomotor triangle; SOF: superior orbital fissure; V1: ophthalmic nerve; V2: maxillary nerve; V3: mandibular nerve; FR: foramen rotundum; FO: foramen ovale; GG: gasserian ganglion; MHT: meningiohypophyseal trunk.

Both CSs were successfully exposed in 12 heads (Figure 1).

**Figure 2:**
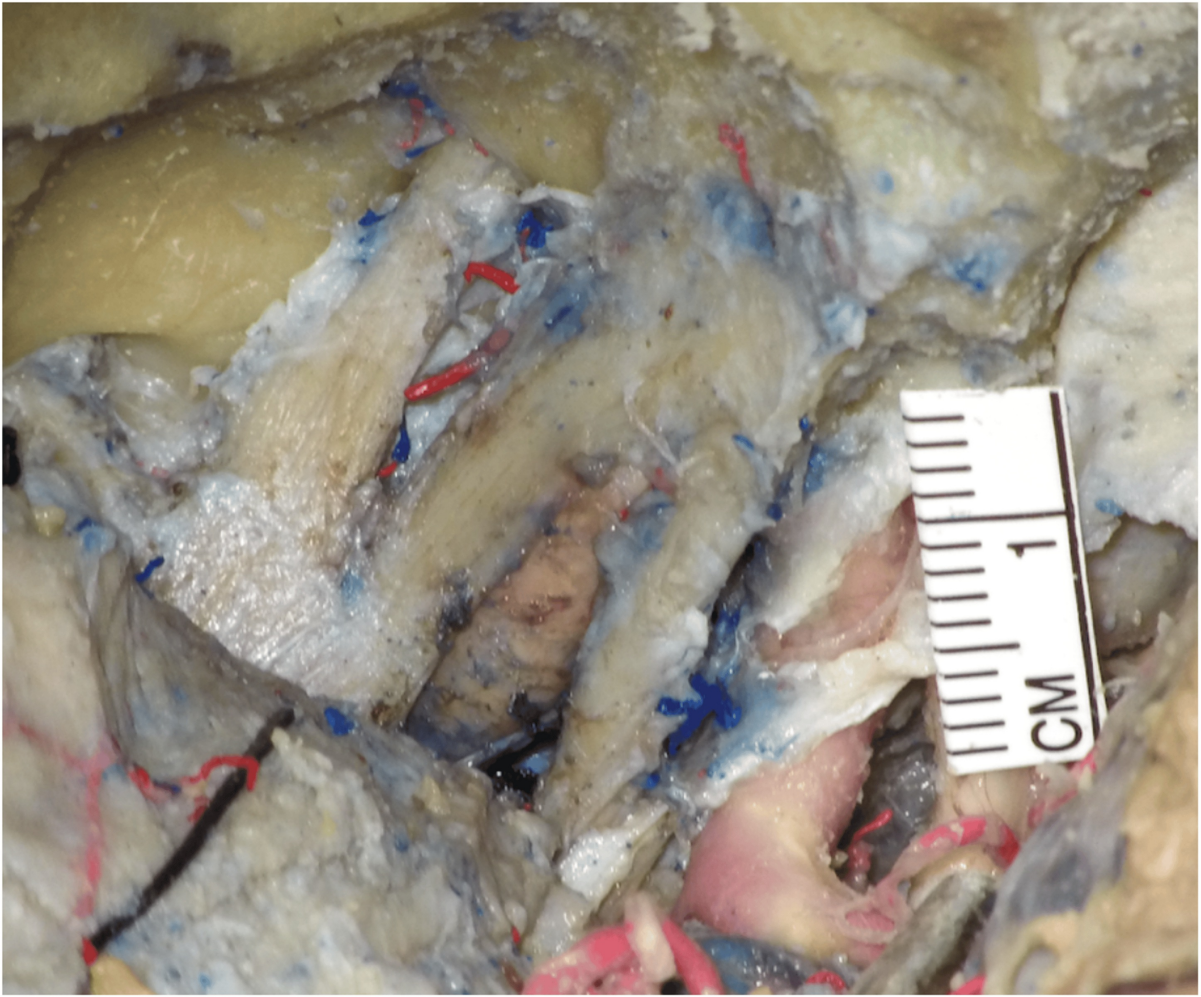
Measurement method for the triangle areas and the lengths of the cavernous internal carotid artery Measurements were taken under the microscope (Carl Zeiss Prescott, Oberkochen, Germany).

The left CS of head number 11 was not well preserved for analysis and was excluded. A total of 25 CSs were prepared to analyze the measurements of the triangles (Figure 2).

**Figure 3:**
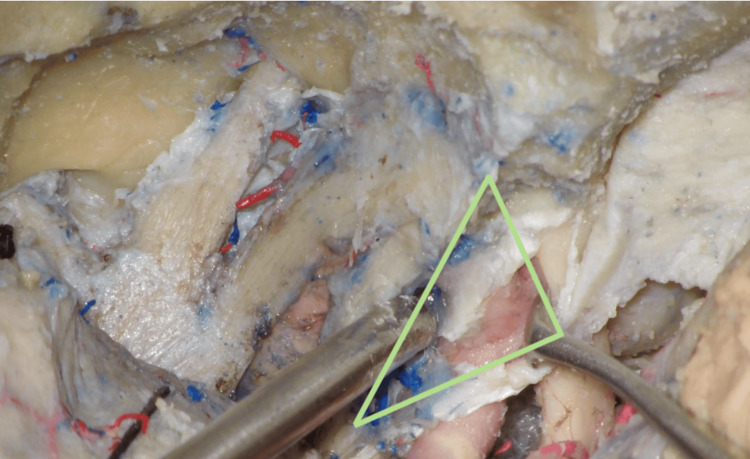
The clinoidal triangle (CT) The triangle limits are the optic nerve medially, the distal dural ring posteriorly, and cranial nerve III laterally. The optic nerve was slightly retracted medially, and cranial nerve III was retracted laterally to expose the internal carotid artery within the triangle.

The CT (Figure 3) was formed between the following structures: (1) the optic nerve medially, (2) the distal dural ring posteriorly, and (3) cranial nerve III laterally.

**Figure 4:**
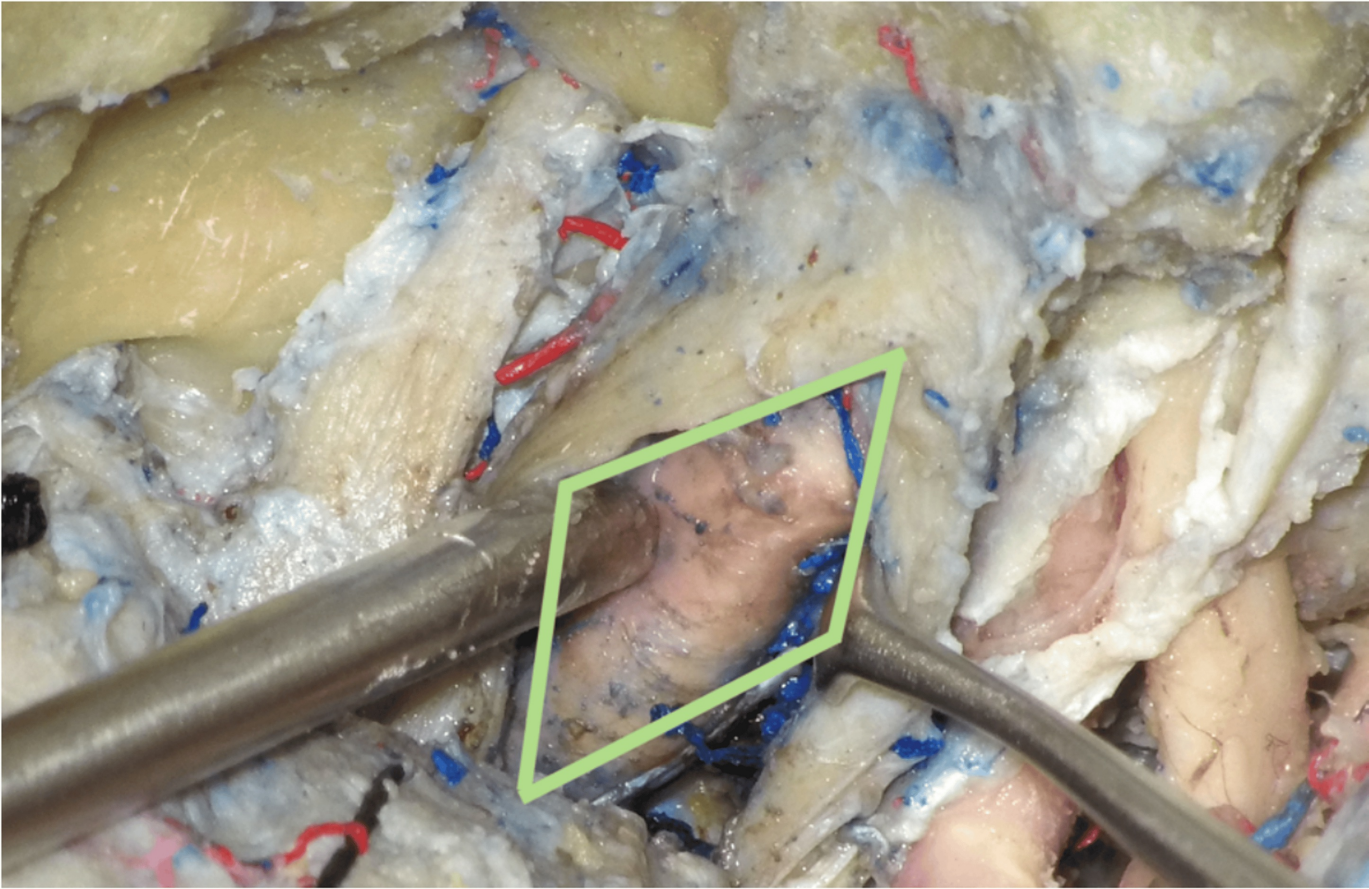
The infratrochlear triangle (ITT) The triangle limits are cranial nerve III medially, cranial nerve IV posteriorly, and the V1 nerve laterally. Cranial nerve III was retracted medially, and the V1 nerve was retracted laterally to expose the internal carotid artery within the triangle.

The ITT (Figure 4) was formed between the following structures: (1) cranial nerve IV posteriorly, (2) cranial nerve III medially, and (3) the V1 nerve laterally.

**Figure 5:**
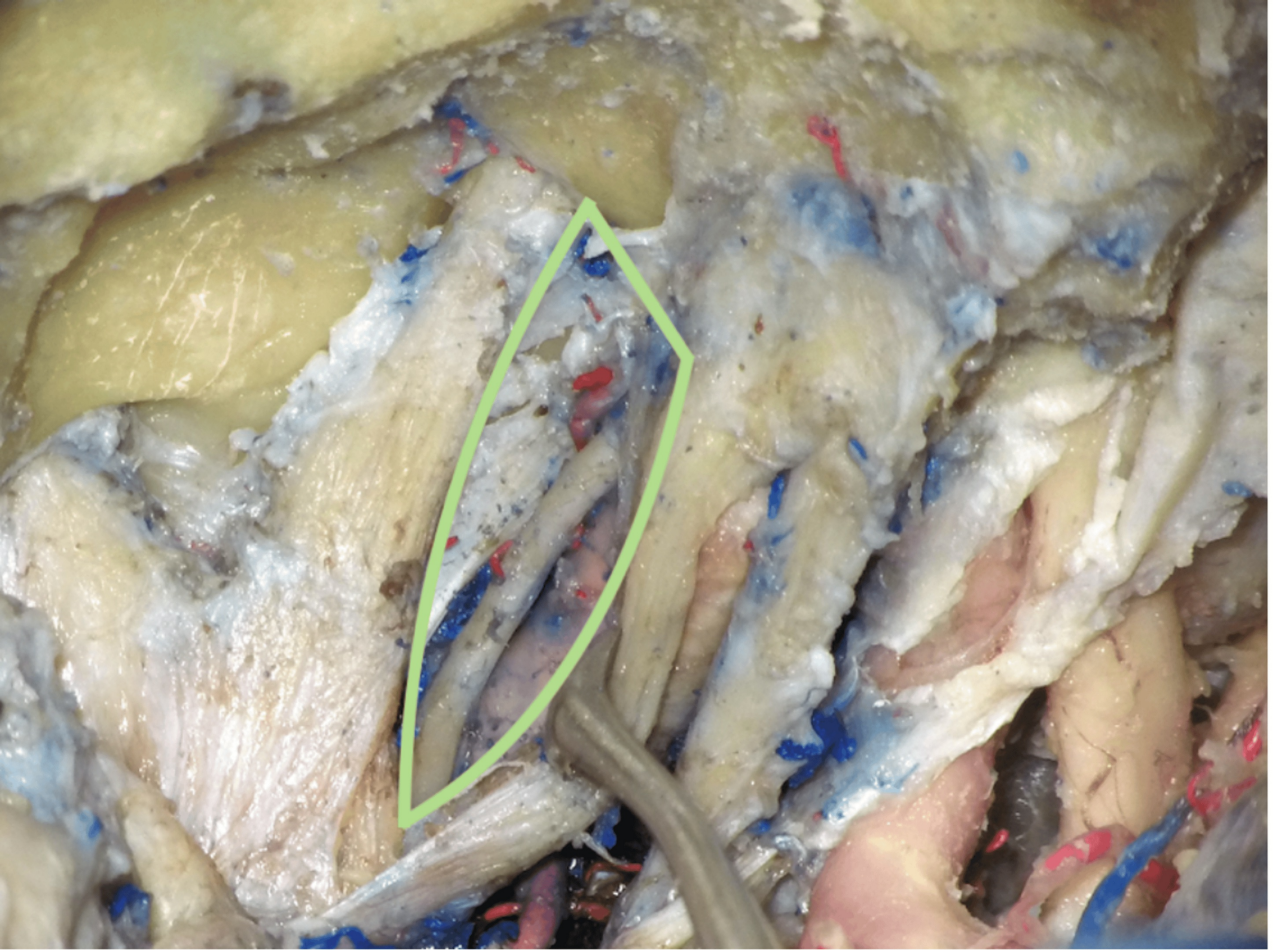
The anteromedial triangle (AMT) The triangle limits are the V1 nerve medially, the V2 nerve laterally, and a line connecting the foramen rotundum and superior orbital fissure anteriorly. The V1 nerve was retracted medially to expose the internal carotid artery within the triangle. The VI nerve was also exposed within the anteromedial triangle.

The AMT (Figure 5) was formed between the following structures: (1) the V1 nerve medially, (2) V2 nerve laterally, and (3) line connecting the foramen rotundum and superior orbital fissure anteriorly.

The results of the triangular areas and lengths of the IC-ICA are listed in Tables 1-3.

**Table 1:** Results of the measurement of CT, ITT, and AMT areas CT: clinoidal triangle. ITT: infratrochlear triangle. AMT: anteromedial triangle. Measurements are presented in mm^2^; ^*^Left CS was not preserved for analysis.

Cadaver	CT left	ITT left	AMT left	CT right	ITT right	AMT right
1	72	64	150	52.3	120	38.7
2	64.9	126	77	62.5	71.9	62
3	27.8	130	43.4	43.3	29.9	66.1
4	35.9	67.7	12.5	69	34.3	32.8
5	35.5	46.9	44.8	62.5	48	41.6
6	58	27.7	35	47.4	35	25.7
7	51.1	52.4	80	29.8	35	60
8	52	34.9	23.7	32.8	52	39
9	30.6	39.9	52.3	95.4	57.8	51.7
10	18.4	31.2	52	36	80.5	59.4
11^*^	-	-	-	36	48	19
12	18.7	47.1	48	19	32.8	16.2
13	24	55.9	45	27.2	39.5	47.2

**Table 2:** Results of the measurement of ICA length exposed through the triangles CT: clinoidal triangle; ICA: Internal carotid artery; ITT: infratrochlear triangle; AMT: anteromedial triangle; Measurements are provided in mm. ^*^Left CS was not preserved for analysis.

Cadaver	CT ICA left	ITT ICA left	AMT ICA left	CT ICA right	ITT ICA right	AMT ICA right
1	7	9	12	21	14	6
2	10	16	12	6	19	14
3	9	15	11	9	6	11
4	9	17	5	6	5	6
5	7	14	5	7	14	12
6	4	11	5	7	4	4
7	7	15	11	8	15	16
8	5	14	3	6	14	6
9	7	5	6	14	10	6
10	7	9	6	7	16	9
11^*^	-	-	-	7	8	3
12	5	6	4	6	8	2
13	4	8	3	7	10	6

**Table 3:** Results of mean triangle areas, IC-ICA exposed lengths, and their statistical comparison CT: clinoidal triangle; ITT: infratrochlear triangle; AMT: anteromedial triangle; IC-ICA: intracavernous internal carotid artery; Measurements are represented as means ± standard deviations; Triangle areas and ICA length are provided in mm^2^ and mm, respectively; ^*^Results of the Kruskal–Wallis test.

	CT	ITT	AMT	p value^*^
Triangle area	44.1 ± 19.6	56.3 ± 29.4	48.9 ± 27.3	0.403
IC-ICA lenght	7.7 ± 3.5	11.3 ± 4.3	7.4 ± 3.9	0.002

The mean area of the CS exposed through the ITT was 56.3 ± 29.4 mm^2^, compared with 48.9 ± 27.3 mm^2^ and 44.1 ± 19.6 mm^2^ for the AMT and CT, respectively. These differences were not statistically significant (p=0.403). However, the length of the IC-ICA exposed through the ITT was significantly greater than the AMT and CT (11.3 ± 4.3 mm vs. 7.4 ± 3.9 mm and 7.7 ± 3.5 mm, respectively) (p=0.002).

## Discussion

The CS is an intricate, deep neurovascular structure that requires extensive anatomical knowledge and microsurgical training. The IC-ICA is considered an inaccessible structure for the direct repair of aneurysms or CCFs. Endovascular techniques play a major role in the treatment of IC-ICA pathology [23-25].

For IC-ICA aneurysms, endovascular interventions have a reported risk of visual and cranial nerve deficits ranging from 6.1% to 38%. For cases with pre-existing compressive optic neuropathy, 38% to 60% of patients showed improvement after the procedure. By contrast, 64% to 92.2% of patients with pre-existing oculomotor nerve palsy experienced improvement, whereas 33% of patients with trigeminal neuralgia showed improvement. The reported occlusion and retreatment rates were 82% and 70.6%, respectively [26-28].

For CCF, the overall success rate of endovascular interventions has been reported to range from 76.6% to 92% [29-33]. However, a substantial recurrence rate of 10% to 28%, a 10.9% complication rate, and overall morbidity and mortality rates of 6% to 10% have also been reported [30,33-35].

Another option is indirect repair through endovascular ICA occlusion following extracranial-intracranial bypass. This method carries the following risks: (1) permanent neurological morbidity, 5.3% to 27.3%; (2) graft occlusion, 2% to 5%; and (3) mortality, 2% [36-41].

There are few publications on the direct microsurgical repair of IC-ICA pathology. Liao et al. [42] reported five illustrative cases of IC-ICA aneurysms treated with clipping via a pretemporal transcavernous approach. They described transient oculomotor and trochlear nerve palsies in four patients, which resolved within 12 months postoperatively. Complete obliteration of the aneurysms was achieved in all cases, and recovery from preoperative diplopia, ptosis, and facial numbness was observed by six months postoperatively. Hakuba et al. [43] reported the outcomes of four vascular lesions in the CS: one aneurysm, two CCFs, and one tentorial arteriovenous malformation. All lesions were successfully treated. Three patients experienced transient oculomotor nerve palsy, and one patient had a permanent deficit. Dolenc et al. [44] reported 93% IC-ICA patency, 2.6% mortality, and 6% permanent cranial nerve III palsy in 115 IC-ICA aneurysms treated using a direct surgical approach. Other authors have reported the safety and efficacy of direct microsurgical approaches for IC-ICA pathologies in small case series [45-47].

Our results support the findings of previous anatomical studies, which have described the ITT as an effective space for reaching the IC-ICA [48,49]. In Parkinson’s pioneering article [2], the ITT was described as “a considerable triangular space between the third and fourth nerves above and the sixth nerve and ophthalmic division of the fifth nerve below”. Dolenc et al. [46] also described the ITT as “the most trustworthy surgical landmark” in a series of seven patients with IC-ICA pathologies successfully treated with direct repair. Previous anatomical studies have described the triangular dimensions as follows [49-54]: ITT, 23.9 to 42 mm²; CT, 19 to 45.6 mm²; and AMT, 18.9 to 56 mm^2^.

Furthermore, the ITT has recently been described as an adequate space for proximal control of the IC-ICA in the clipping of complex paraclinoid aneurysms [12]. To the best of our knowledge, no studies have conducted a morphometric comparative analysis of CS triangles and the exposed IC-ICA.

Our findings provide further details regarding the microsurgical anatomy of the CS and its access triangles to the IC-ICA. These results could be applied to direct repair of IC-ICA pathology instead of relying solely on endovascular or anastomotic procedures. 

Further studies using larger samples, different fixation techniques, and additional measurement tools are needed to confirm these findings.

## Conclusions

In this anatomical study, the microsurgical anatomy of the CS and its access triangles to the IC-ICA were carefully examined. The ITT was identified as the most suitable space for reaching the IC-ICA compared to the CT and AMT. It can be widely exposed using a pretemporal transcavernous approach. This surgical window can be used to directly repair various IC-ICA pathologies, such as aneurysms or CCFs. Nevertheless, the potential value of a direct approach to the IC-ICA must be carefully studied further.
